# Costs of Rabies Control: An Economic Calculation Method Applied to Flores Island

**DOI:** 10.1371/journal.pone.0083654

**Published:** 2013-12-27

**Authors:** Ewaldus Wera, Annet G. J. Velthuis, Maria Geong, Henk Hogeveen

**Affiliations:** 1 Animal Health Study Program, Kupang State Agriculture Polytechnic, West Timor, Indonesia; 2 Business Economics Group, Wageningen University, Wageningen, The Netherlands; 3 Husbandry Department of East Nusa Tenggara Province, Indonesia; Thomas Jefferson University, United States of America

## Abstract

**Background:**

Rabies is a zoonotic disease that, in most human cases, is fatal once clinical signs appear. The disease transmits to humans through an animal bite. Dogs are the main vector of rabies in humans on Flores Island, Indonesia, resulting in about 19 human deaths each year. Currently, rabies control measures on Flores Island include mass vaccination and culling of dogs, laboratory diagnostics of suspected rabid dogs, putting imported dogs in quarantine, and pre- and post-exposure treatment (PET) of humans. The objective of this study was to estimate the costs of the applied rabies control measures on Flores Island.

**Methodology/principal findings:**

A deterministic economic model was developed to calculate the costs of the rabies control measures and their individual cost components from 2000 to 2011. The inputs for the economic model were obtained from (i) relevant literature, (ii) available data on Flores Island, and (iii) experts such as responsible policy makers and veterinarians involved in rabies control measures in the past. As a result, the total costs of rabies control measures were estimated to be US$1.12 million (range: US$0.60–1.47 million) per year. The costs of culling roaming dogs were the highest portion, about 39 percent of the total costs, followed by PET (35 percent), mass vaccination (24 percent), pre-exposure treatment (1.4 percent), and others (1.3 percent) (dog-bite investigation, diagnostic of suspected rabid dogs, trace-back investigation of human contact with rabid dogs, and quarantine of imported dogs).

**Conclusions/significance:**

This study demonstrates that rabies has a large economic impact on the government and dog owners. Control of rabies by culling dogs is relatively costly for the dog owners in comparison with other measures. Providing PET for humans is an effective way to prevent rabies, but is costly for government and does not provide a permanent solution to rabies in the future.

## Introduction

Rabies is a zoonotic viral disease caused by a member of the *Lyssavirus* genus in the *Rhabdoviridae* family [1], [2]. The main transmission route to humans is through animal bites, especially those of dogs [3]. In humans, the virus infects the peripheral nerves and spreads to the brain (central nervous system), resulting in encephalomyelitis [4] and hydrophobia, which is the most specific clinical sign of rabies [3]. Once clinical signs appear, fatality is almost 100 percent [5]. The World Health Organization [6] estimated that 55,000 people die each year due to rabies around the world, with over 99 percent of these cases occurring in Africa and Asia [7]. In Indonesia, 150–300 fatal cases of human rabies are reported annually [8], with approximately 19 on Flores Island [9] where dogs are the principal reservoir for transmitting the virus to humans [10].

Control of rabies in dogs is an important means to prevent rabies in humans. Possible control measures include mass vaccination of dogs, culling roaming dogs, quarantining imported dogs, and movement restrictions of dogs. Vaccination of dogs offers a safe and effective means to control rabies as has been reported for some endemic countries [11], [12], [13], [14]. The first successful example of a mass vaccination program in a dog population occurred in the city of Memphis and Shelby County, Tennessee in the United States in 1948 [11]. The number of rabies cases in both animals and humans was reduced to zero [11]. Success stories were also reported from Latin American countries, where mass vaccination of the dog population has led to reduction of rabies in humans [12]. More recently, mass vaccination of dogs in Tanzania [13] and Bali Island, Indonesia [14] successfully decreased dog and human rabies cases. Other control measures than vaccination enabled the United Kingdom to become free of rabies in 1922. These measures included shooting stray dogs, strict muzzling of all pet dogs, and quarantining imported dogs [15], [16]. Measures to reduce the burden of rabies in humans include pre-exposure treatment (vaccination of human at risk before exposure) and post-exposure treatment (wound cleaning, immunoglobulin injection, and series of vaccine injections after bitten by a suspected rabid dog) [17].

Rabies is a costly disease [17] mainly because of the costs of post-exposure treatment (PET) in humans and vaccination programs in animals. PET in humans accounts for the highest proportion of the costs of rabies control measures. Knobel et al. [18] reported that 83 percent (US$485 million) of the total rabies control budget in Asia and Africa was allocated to PET. The costs of PET include costs for rabies immunoglobulin and vaccines and for physician and hospital services [19]. Vaccination costs in animals vary among countries, depending on the epidemiological features of the disease. For example, the annual costs of animal rabies vaccination were estimated to be US$5.5 million in Canada [20] and US$ 4.1 million in the Philippines [21].

Located in eastern Indonesia, Flores Island is populated by over 1.8 million humans [22] and 236,500 dogs (as registered by the Husbandry Department of East Nusa Tenggara Province in 2011). The first officially confirmed case of rabies appeared in 1998 when dogs with the disease were imported from Sulawesi Island. The response was total culling of all dogs [9], [23]. Unfortunately, this control measure failed to stop the spread of the rabies virus. Therefore, in 2000, the Flores Island government implemented a combination of control measures, including mass vaccination of dogs, culling of roaming dogs, placing imported dogs in quarantine, and giving pre- and post-exposure treatment to humans. In addition, complementary control measures were applied, such as dog bite investigation, diagnostic testing of suspected rabid dogs, and trace-back of human contacts with rabid dogs.

Although there are some economic evaluations of rabies outbreaks published for South and South East Asia [21], [24], [25], [26], [27], none of these publications were dedicated to the situation of rabies in Indonesia and none of these publications described an integral economic evaluation of rabies control, taking into account the costs of control measures both in dogs and humans for different stakeholders (i.e. Animal Health Department, dog owners, dog-bite patients and Public Health Department). Therefore, this study sought to calculate the costs of the rabies control measures both in dogs and humans (with specified costs of rabies control measures for different stakeholders and the costs of culling roaming dogs) applied on Flores Island since 2000. The results of this study provide insights which are useful for decision makers who need to decide upon the rabies control programs in the future.

## Materials and Methods

An economic model was developed using Microsoft Excel 2010 to evaluate the costs of various rabies control measures and the distribution of the costs among the various stakeholders on Flores Island. The inputs for the economic model were obtained from: (i) relevant scientific literature, (ii) available data on Flores Island, and/or (iii) experts such as responsible policy makers and veterinarians involved in rabies control measures on Flores Island. The values of the input obtained from scientific literature were related to the indicated year of the described study or, if not present, to the year of publication. The cost in different years (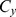) was compounded to 2011 (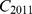) using the following formula:
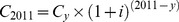
(1)


Where, 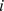 is the discount rate which was set at 6% [28] and 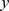 is the year in which the costs were made. Costs involved in each control measure were converted into US dollars, using the currency rate on January 31, 2012 which was US$1 = Rp 9045 (http://www.bi.go.id). A sensitivity analysis was performed using add-in software TopRank 6.0 for Excel of Palisade Decision Tools to identify the inputs that were highly influential to the output. Furthermore, the costs of each measure were ranked based on their contribution to the total costs.

### Economic Model

A deterministic economic model was built to evaluate the total costs of control measures 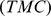 applied both in dogs and humans:
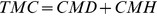
(2)


Where, 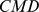 represents the costs of control measures in dogs, and 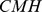 represents the costs of control measures in humans.

### Control Measures in Dogs

The total costs of rabies control measures in the dog population equal the sum of the costs of six control measures: (i) mass vaccination 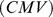, (ii) culling of roaming dogs 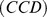, (iii) dog-bite investigations 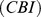, (iv) diagnostic testing of suspected rabid dogs 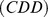, (v) trace-back investigation of human contacts with rabid dogs 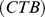, and (vi) quarantining of imported dogs 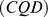:
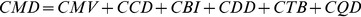
(3)


In the following paragraphs, each control measure in the dog population is explained and detailed economic calculations are given for each, including the inputs.

#### Mass vaccination of dogs

A rabies vaccination program that is free of charge and compulsory for all dog owners has been in effect in the Ende and Manggarai regencies of Flores Island since 2000 [9]. In 2001, the program was expanded to other regencies, namely, East Flores, Sikka, Nagakeo, East and West Manggarai. Several activities are involved to make the vaccination campaign operational, including organization, communication, and vaccination activities.

The organizational activities include planning the campaign, recruitment and training of temporary vaccinators, and selection activities of the areas. The planning began with a meeting to determine the vaccinators, the budget, and the distribution of campaign information. The vaccinators were veterinary assistants graduated from an animal health and/or a husbandry study program at a university or senior high school. To increase vaccination coverage, a veterinarian occasionally trained local people and community nurses as temporary vaccinators, as in 2008. The Agricultural Department in each regency incurred the available budget for the campaign.

The communication activities included development and distribution of materials to inform the local community about the vaccination campaign and to stimulate dog owners to vaccinate their dogs. The campaign information was sent to the heads of the villages, religious leaders, and a radio station, and/or was broadcasted from a car with a loudspeaker once a week before the mass vaccination began. The head of each village was asked to encourage dog owners to bring their dogs to a designated place and/or to confine at home for the vaccinator. Religious leaders were asked to announce the campaign schedule in churches and mosques. The radio station was asked to make announcements on consecutive days before the campaign began. Additionally, leaflets and posters were distributed in public areas.

The vaccination activities included the vaccination of dogs and an educational program for the local community. On the day of the mass vaccination, vaccinators, veterinarians, and staff of the Regency Agricultural Department went to rural and urban areas to vaccinate dogs and to educate the local community to keep dogs under supervision. Vaccinations were delivered by subcutaneous administration and required a booster at three months to confer one year’s protection [29]. The vaccine used was Rabivet Supra® (Pusvetma, Surabaya, Indonesia). Sometimes, depending on the allocated budget, a vaccinated dog was collared with a wire collar and tag [23]. The total number of registered dogs vaccinated on the island was on average 53 percent (range: 23–82 percent) of the total registered dog population during the vaccination campaign (Table 1).

**Table 1:** Total number of registered dogs (*n*), vaccinated dogs (*n_vd_*), culled dogs (*n_cd_*), samples submitted (*n_ss_*), and tested positive (*n_sp_*) in Flores Island from 2000 to 2011.

	Number of dogs			Number of samples	
Year	Total (*n*)	Vaccinated (*n_vd_*)	Culled (*n_cd_*)	Submitted (*n_ss_*)	Positive (*n_sp_*)
2000	213,004	49,632	27,050	1,935^a^	1,550
2001	165,411	50,297	25,181	946^a^	760
2002	165,411	79,058	25,297	279	219
2003	169,035	126,343	4,312	31	13
2004	207,099	168,921	9,988	30	13
2005	250,372	172,763	14,697	26	7
2006	260,269	142,903	16,183	12	9
2007	201,322	78,086	22,603	10	9
2008	236,378	146,155	12,836	3	2
2009	257,841	158,086	5,436	7	3
2010	233,739	130,637	234	28	15
2011	236,447	78,231	106	39	28

Source data: Husbandry Department of East Nusa Tenggara Province. These data were registered by each Regency Husbandry Department in Flores Island as part of vaccination campaign. In case the dog owners and their dogs were not present at time of registration, the dogs were not accounted for. For example in Sikka regency, the dogs of approximately 30% of the dog owners were not registered for this reason in 2012 (Personal communication, Dr. Sikko). As a result the registered number underestimates the actual size of the dog population.

^a^ Windiyaningsih et al., [9].

The costs of mass vaccination 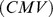 include costs of the vaccine 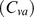, costs of consumables, such as needles, syringes, etc. 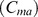, costs of vaccinators 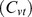, costs to train the temporary vaccinators 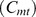, costs of the information campaign 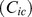, capital costs 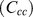, and opportunity costs for the time of the dog owners to catch and restrain their dogs for vaccination 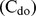:
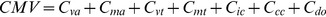
(4)



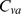
 depends on the price of the vaccine per dose 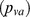, costs of transportation of the vaccine from manufacturer to each regency 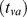, and the number of registered vaccinated dogs 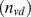:
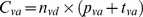
(5)



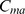
 depends on the price of needles and syringes 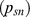, ice bars 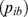, disinfectant swabs 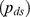, the proportion dogs using collar after vaccination 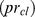, and the price of collar 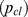:
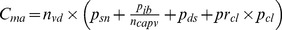
(6)


Where, 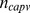 is the average number of registered dogs vaccinated by one vaccinator per day.

The vaccination of dogs was administered by a group of temporary vaccinators under close supervision of a veterinarian or public servant. Therefore, costs of vaccinators 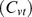 consist of the costs for temporary vaccinators 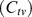 and costs for public servants who supervise the vaccinators 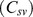:
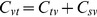
(7)


Where, 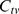 consists of the number of registered vaccinated dogs multiplied with the salary 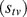 and fuel costs (per day) for travelling 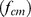 of the vaccinator per day :
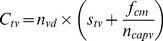
(8)



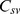
 was calculated based on the number of vaccination days 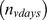, the costs per day per public servant or veterinarian 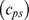 and the fuel costs for travelling 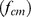 per day:
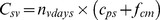
(9)


The number of vaccination days depends on the number of vaccinators who can be supervised by one public servant 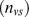:
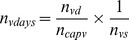
(10)



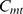
 includes costs of meeting and training of temporary vaccinators while 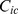 includes costs of printing and distribution of the leaflets and posters, and the development and broadcast of the radio advertisements. 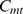 and 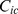 were not calculated, but were given as a fixed budget item reported by a government veterinarian responsible for the rabies control program (2012, personal communication).


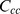
 includes the yearly depreciation costs for cool bags, refrigerators, motorcycles, and muzzles:
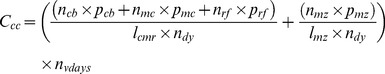
(11)


Where 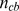 is the number of cool bags needed each year, 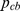 the price of a cool bag, 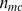 the number of motorcycles, 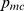 the price of a motorcycle, 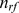 the number of refrigerators, 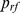 the price of a refrigerator, 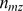 the number of muzzles, 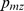 the price of a muzzle, 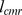, 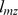 the number of life years of capital goods (cool bags, motorcycles, and refrigerators) and muzzles (expected to be used in any diseases control programs), and 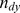 the number of days in a year. Note that 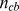, 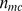, 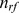, and, 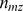 increased with the number of new villages to be vaccinated [9]; however, for simplification, the average numbers for Flores Island were used for each year. We assumed the salvage value of capital goods and muzzles to be equal to zero.


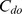
 was calculated based on the opportunity cost for the dog owner’s time to catch and restrain a dog 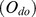 and the number of vaccinated dogs:
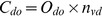
(12)



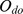
 was based on the number of working hours lost per dog owner 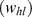, the average daily wage of a dog owner 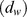, and the number of hours work per day 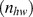:
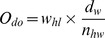
(13)


As the vaccination campaign was conducted during working days when the children were at school, we assumed that all the handlers of dogs during the campaign were adult people.

The inputs used in the calculations for the costs of mass vaccination in dogs are presented in Table 2. The calculation of the mass vaccination campaign was based on a door-to-door approach as most vaccination programs in Flores Island were carried out as door-to-door campaigns (2013, Personal communication).

**Table 2:** Model inputs for the cost calculations of control measures in dogs (Prices expressed at level of 2011).

Description	Variable		Value (Rp)	Value (US$)	Unit
**Mass vaccination**					
Price of vaccine	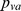		2,631^ola^	0.29	Rp/dose
Transportation costs of vaccine from manufacturer to each regency	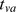		1,390^bgb^	0.15	Rp/dose
Price of syringes and needles	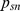		1,750^ggb^	0.19	Rp/dog
Ice bars	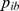		3,000^fffb^	0.33	Rp/coolbag/day
Vaccination capacity	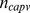		25^c^		Dogs/vaccinator/day
Disinfectant swabs (70% ethanol or alcohol) for cleaning the dog’s skin	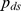		200^b^	0.02	Rp/dog
Proportion of vaccinated dogs using collar	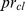		10%^b^		
Price of collar	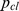		3,000^b^	0.33	Rp/piece
Salary of temporary vaccinator	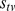		2,500f,hb	0.28	Rp/vaccinated dog
Transportation cost for people involved in the rabies control	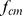		9,000^ggd^	1.00	Rp/person/day
Costs of public servant	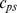		91,000^e^	10.06	Rp/person/day
The number of vaccinators that can be supervised by one public servant	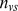		10^b^		vaccinators/supervisor
Costs of training and meeting	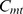		7,700,000^b^	851.30	Rp/year
Campaign costs	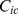		120,000,000^b^	13,267	Rp/year
Cool bags	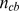		27^f^		Pieces
Price cool bag	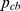		253,170^g^	27.99	Rp/piece
Motorcycles	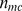		16^f^		Pieces
Price motor cycle	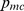		15,100,000^h^	1,669	Rp/piece
Refrigerator	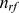		8^f^		Pieces
Price refrigerator	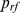		1,580,000^h^	174.68	Rp/piece
Muzzles	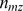		27^f^		Pieces
Price of muzzle	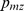		50,000^h^	5.53	Rp/piece
Life years of capital goods (cool bags, refrigerators and motorcycles)	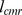		5^i^		years
Life years of muzzles	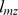		2^b^		years
Number of days in one year	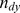		365^i^		days
Working hours lost for a dog owner	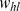		2^j^		Hours/vaccinated dog
Daily wage	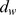		39,000^k^	4.31	Rp/day
Number hours work	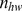		8^i^		Hours/day
**Culling of roaming dogs**					
Value of dogs	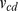		278,923^l^	30.80	Rp/dog
Proportion of dogs culled by local community or dog owners	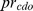		80%		
Opportunity cost of time to cull dogs for local community or dog owners	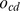		2,500^m^	0.28	Rp/person/dog
The number of dogs that can be culled by a governmental team	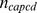		40^b^		dogs/team/day
Price of ammunition (bullet)	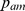		9,241^n^	1.02	Rp/bullet/dog
**Dog-bite investigation**					
Number of investigators	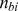		1^o^		Person/case
Cost of the investigators	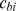		191,000^e^	21.12	Rp/investigator
Material costs (gloves, scissors, and tweezers)	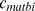		7,000^b^	0.77	Rp/sample
**Diagnostic testing of suspected rabid dogs**					
Material costs (glycerin, formalin)	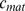	5,000^b^		0.55	Rp/sample
Laboratory costs	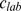	20,000^p^		2.21	Rp/sample
Packing	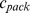	10,000^b^		1.11	Rp/sample
Shipping	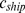	20,000^b^		2.21	Rp/sample
Cost of collector sample	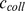	15,000^b^		1.66	Rp/sample
Correspondence of laboratory result	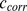	30,000^i^		3.32	Rp/sample
**Trace back investigation of human contacts with rabid dogs**					
Number of people that are doing trace back investigation	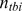		1^o^		person/case
Costs of investigator	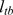		191,000^b^	21.12	Rp/day/investigator
**Quarantine**					
Number of dog quarantined	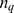		4^q^		dogs per year
Length of quarantine	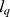		14^r^		days
Cost of quarantine facility	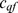		1,500^a^	0.17	Rp/day/dog
Cost of dog food	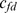		5,000^i^	0.55	Rp/day/dog
The quarantine caretaker salary	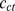		2,500^a^	0.28	Rp/day/dog
Cost of veterinary inspection	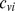		7,500^a^	0.83	Rp/period quarantine/dog
Cost of administration (sertificate document, ect)	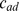		7,500^a^	0.83	Rp/period quarantine/dog

^a^ Indonesian Agriculture Ministry (IAM) [53]);

^b^ Public servants/veterinarians involved in rabies control measures in the past;

^c^ Vaccinators involved in the vaccination campaign;

^d^ Calculated: Multiplying by the average distance between the vaccination location and the Regency Agricultural Department (in average 100 km, rate of fuel consumption (in average 1litter per 50 km [54]) and market price of fuel per litter (Rp 4,500 per litter).

^e^ The real cost paid to a public servant (Rp 100,000 per person per day) minus his/her transportation cost (Rp 9,000 per person per day);

^f^ Average number based on data from Husbandry Department of Sikka and Ngada regencies;

^g^ http://www.igloo-store.com/detail/IGLDUOSTCOOLG (accessed 24 June 2013);

^h^ Market price in Flores by asking the seller in the shopping center;

^i^ Assumption based on the author knowledge;

^j^ Dog owners participated in the vaccination program;

^k^ BPS (Indonesian Statistics) [55];

^l^ Calculated based on the average value of dogs year 2003, Rp 175,000 per dog (Hutabarat et al., [23]);

^m^ Calculated based on the daily wage and the number of dog culled per day per person (approximately 16 dogs per day per person);

^n^ Michell and Kanowski [56];

^o^ Husbandry Department of Sikka Regency;

^p^ Center of Disease Investigation, Maros;

^q^ Ende Regency quarantine;

^r^ Indonesian quarantine (IQ) [57];

#### Culling of roaming dogs

According to [9], [10], [23], it is unlikely there are ownerless dogs in Flores Island. Majority of the dogs is unrestrained and allowed to roam freely, hence the term free-roaming dogs. The decision to cull roaming dogs was generally considered in one of the following three situations: (1) when the virus was newly introduced into an area, all dogs in that area would be culled; (2) when a dog was freely roaming in a public place regardless of its vaccination status; and (3) when an unvaccinated dog was freely roaming in a public place.

The diagnosis of whether the virus was newly introduced in an area was based on the occurrence of clinical signs in a human who lived in that area, accompanied by test results of suspected dogs in that area. In this case, the regency administrator released a warning regarding the rabies danger, usually followed by mass dog culling in that area. For example, when rabies was introduced to East Flores Regency in 1998 and to Ngada Regency in 2000, each regency administrator decided to cull all dogs throughout the regency [30].

Culling any dog freely roaming in a public place, regardless of vaccination status, has been applied in Manggarai Regency (Manggarai Regency’s law number 6, year 2003). Public places include roads, public parks, traditional markets, and open fields.

Culling unvaccinated dogs freely roaming in public places was initiated in Ngada Regency in 2001, and expanded into all other regencies on Flores Island except for Manggarai Regency. This program was not operating well because of a lack of regulation to force people to comply. The culling program was carried out in collaboration between government and local community, and was conducted within villages during the day light by shooting (generally by a team that formed by regency administrator) or by beating the dogs with a stick (by local community). The majority of the culling was carried out by the local community and dog owners themselves [23]. Since actual data is lacking, we assumed only 20% of the total culled dogs to be executed by a governmental team (based on the experiences of the local veterinarians involved) which included a public servant and police or army assistance.

The cost of culling roaming dogs 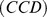 includes private costs 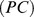 and public (governmental) costs 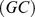:
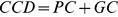
(14)



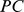
 only depends on the number of dogs culled (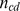), the value of dogs (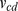), and the proportion by which the dogs are culled by the local community 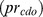, and the opportunity cost for their time investment to cull one dog 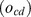:
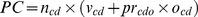
(15)



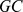
 includes the costs per day per governmental team culling dogs (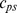), the price of a bullet used to shoot a dog (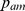), the fuel costs per day of the team 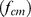, and the daily depreciation cost of the motorcycles needed to travel to the culling area (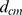) :
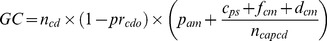
(16)



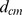
 was calculated based on the number of motorcycles 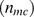, the price of a motorcycle 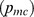, and the number of life years of motorcycles 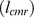:
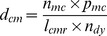
(17)


Depreciation costs of guns and sticks were ignored since these were negligible. The guns were provided by police and army departments and were not special purchased for shooting dogs. The sticks were already available in the village.

The costs for an information campaign regarding culling dogs are included as an integral part of the campaign of the mass vaccination program. The inputs used in the calculations for the costs of culling control measures are presented in Table 2. In addition, the total number of dogs culled per year on Flores Island is shown in Table 1, which was on average 7 percent (range: 0–15 percent) of the total registered dog population during the vaccination campaign.

#### Dog-bite investigation

When a bite from a suspected rapid dog was reported, the veterinary authority (investigators) gathered information from the victim and the dog owner. Officially, the veterinary authority would capture and quarantine the dog for 10 days, but usually the owner or the victim’s family already had killed the dog. In this context, the veterinary authority would collect a brain sample or a head of dog to be sent to the central laboratory in Maros, Sulawesi.


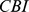
 includes costs of the investigators who were involved in the investigation of the biting case 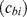, costs of materials, such as gloves, scissors, and tweezers 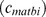, and costs of transportation for the investigators (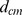 and 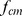):
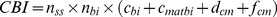
(18)


Where, 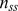 is the number of samples submitted and 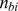 the number of investigators involved in the investigation. The diagnostic costs are explained in the following paragraph. The inputs for this calculation are given in Table 2, and the number of dogs investigated in Table 1.

#### Diagnostic testing of suspected rabies dogs

Diagnostic testing is an integral part of the control program to obtain accurate incidence data. Therefore, all suspected rabies cases in dogs should be confirmed by clinical samples that are tested at a diagnostic laboratory [31] using fluorescent antibody test [32]. In total 2,988 samples from suspected rabid dogs from Flores Island were sent to the laboratory in Maros, South Sulawesi for rabies testing from 2000 to 2011. These samples came from dogs that bit humans, as mentioned in the dog bite investigation activity. All samples were sent by postal services, and results were sent by postal service to the Animal Health director in Jakarta, the head of the Animal Husbandry of East Nusa Tenggara Province (in Kupang), and the head of the Regency Agricultural Department in Flores Island.

The total costs of testing suspected rabies dogs depend on the number of samples submitted, transported, and tested and the corresponding cost of the results:
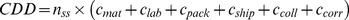
(19)


Where, 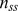 is the number of samples submitted to the laboratory, 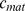 the costs of materials, such as glycerin and formalin, 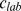 the laboratory costs, 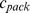 the costs for packing the samples; 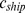 the shipping costs, 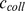 the costs for collection of samples (or sampling activity), and 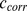 the cost for correspondence of laboratory results.

The inputs of these calculations are listed in Table 2. The number of samples tested in 2000 and 2001 was high, relative to later years because there were severe outbreaks in Ngada and Manggarai Regencies with more than 1,894 and 712 bite cases in 2000 and 2001, respectively.

#### Trace back investigation of human contacts with rabid dogs

When a brain sample of a suspected dog tested was positive for rabies, the authorities attempted to trace all persons who may have had contact with the dog. Anyone bitten by the dog was vaccinated.

The costs for tracing back the human contacts of rabid dogs 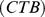 include transportation costs of the person doing the work (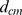 and 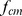) and the labor costs of this person 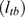:
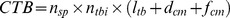
(20)


Approximately, 80 percent of the brain samples tested in the laboratory tested positive for rabies. We assumed that all dogs testing positive 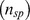 were traced back so that the people who may have had contact with these dogs were investigated. The inputs for this calculation can be found in Table 2.

#### Quarantine of imported animals

The Indonesian government applies a quarantine program of minimal 14-days to prevent reintroduction of rabies through the import of vectors such as dogs, cats, and monkeys to Flores Island.

The quarantine costs 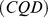 are described as:
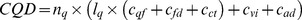
(21)


Where 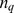 represents the number of dogs quarantined, 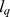 the length of the quarantine period, 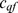 the cost of quarantine facility per day, 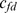 the cost of dog food per day, 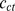 the caretaker salary per dog per day, 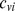 the costs of veterinary inspection per dog per period quarantine, and 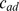 the costs of quarantine administration or document per dog per period quarantine. The input values can be found in Table 2.

### Control Measures in Humans

The total costs of rabies control measures in humans equal the sum of the pre-exposure treatment costs 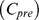 and the PET costs 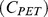:
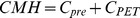
(22)


Each control measure in humans is explained below and a detailed economic calculation is given, including the inputs.

#### Pre-exposure treatment in humans

Pre-exposure treatment is effective to prevent rabies in persons who have a high risk of contact with the virus, such as veterinarians, veterinary assistants, laboratory workers and public servants involved in the rabies control program [6]. The treatment consists of three doses of a rabies vaccine (Verorab®), which is administered prior to the person’s exposure to a suspected rabid dog. The vaccine is administered intramuscularly or intradermally on days 0, 7, and 21 or 28 [6]. If the serological status is below 0.5 IU/ml, a booster after one year is recommended.


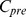
 depends on the number of people at risk that received pre-exposure treatment 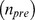, the number of doses of vaccine for pre-exposure treatment 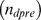, costs of the vaccine 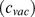, costs of materials such as needles, syringes, and disinfectant swabs (70% ethanol or alcohol) 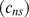, physician costs 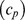 and transportation costs to take high-risk people to and from a hospital to receive the vaccination 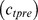:
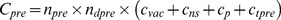
(23)


We assumed that there were no opportunity costs for the public servants who received pre-exposure treatment, since expected time needed to provide a vaccination was less than 1 hour per person. The input values of pre-exposure treatment are given in Table 2.

#### Post-exposure treatment in humans

Post-exposure treatment, which is given to persons bitten by a suspected rabid animal, consists of wound cleaning, one dose of immunoglobulin, and four (Zagreb schedule) or five doses (Essen schedule) of vaccine [33].

The wound should be cleaned with soap for 15 minutes and antiseptic should be used to reduce the contamination from microorganisms [34]. Proper wound cleaning can remove the virus before it spreads to the nervous system, and consequently, the probability of human infection may be reduced [35]. In addition, wound cleaning is sometimes the most feasible option for bitten persons in remote areas; Flores Island has only five regency hospitals that provide vaccine and immunoglobulin treatments, and these may be too far for some individuals to travel.

A rabies immunoglobulin injection around the wound is an essential part of the PET because it neutralizes the virus before it invades the nervous system [36]. Human rabies immunoglobulin (HRIG) is administered only once (at the beginning of anti-rabies prophylaxis) to previously unvaccinated persons to provide neutralizing antibodies immediately [33]. This treatment is highly recommended for those with severe wounds [6].

Vaccination of persons bitten by a suspected dog aims to prevent clinical signs of rabies, and delay contributes to post-exposure treatment failure [37]. Therefore, the vaccination should be applied immediately after exposure [6].

The Indonesian Health Ministry (IHM) [38] recommended using the Zagreb schedule for post-exposure treatment, with four doses injected intramuscularly in three visits on days 0, 7, and 21. On the first visit (day 0), a patient is injected with two doses of vaccine. Then, additional doses are applied on days 7 and 21. The vaccine used in Flores Island was a rabies vaccine produced on Vero cells (Verorab®).


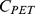
 depends on the costs of wound cleaning 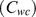, immunoglobulin injection 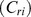, and a series of vaccine injections 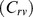:
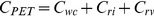
(24)



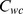
 consists of costs of water 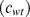, soap 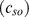, and antiseptic 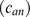 multiplied by the number of persons bitten by a suspected dog 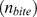:
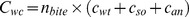
(25)


We assumed that all people bitten by suspected rabid dogs cleaned their wound with water and soap for 15 minutes in line with the general recommendation.

The costs related to rabies immunoglobulin injection 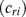 are costs of immunoglobulin 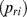, needles, syringes, and disinfectant swabs 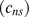:
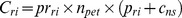
(26)


Where, 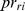 is the proportion of people who received rabies immunoglobulin, and 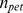 the number of people who received rabies vaccine after exposure to a suspected rabid dog. We assumed no additional costs for transport and physicians since the immunoglobulin injection was performed along with the first injection of vaccine series.

The factors associated with the costs of vaccine injection 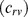 are the cost of vaccine 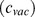, costs of needles, syringes, and disinfectant swabs 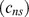, physicians’ fees 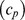, and the number of doses of vaccine for PET 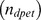, proportion of adult people received PET 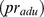, transportation costs 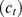 to and from medical center for each dose of vaccine for 2 persons as we assumed that all patients were accompanied by one person:
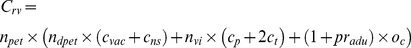
(27)


Where, 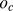 is the opportunity costs of the time of adult patients and one additional person who accompanies the patient to receive a treatment from a nurse or physician. The opportunity costs were calculated based on the daily wage 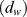 and the number of loss working time during the vaccine series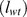:
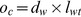
(28)


Table 3 shows the number of humans bitten by suspected dogs and the number of persons receiving PET. All other inputs are shown in Table 4.

**Table 3:** The number of bitten human by rabies suspected dogs and post exposure treatments (PET) in Flores Island during 2000–2011.

Year	Bite cases (*n_bite_*)	PET (*n_pet_*)	Percentage of PET (%)
2000^a^	2,560	1,821	71
2001^a^	1,143	419	37
2002^a^	718	710	99
2003	967	840	87
2004	1,222	1,061	87
2005	3,073	2,668	87
2006	2,231	2,164	97
2007	3,261	3,020	93
2008	3,448	3,011	87
2009	3,764	3,248	86
2010	4,888	3,743	77
2011	3,563	2,889	81

Source data: Human Health Department of East Nusa Tenggara Province.

^a^ Windiyaningsih et al., [9].

**Table 4:** Model inputs for the cost calculations of control measures in humans.

Description	Variable	Value(Rp)	Value(US$)	Unit
Number of people received pre-exposure treatment	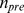	150^a^		Person/year
Number of doses of vaccine for pre-exposure treatment	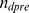	3^b^		Doses/patient
Cost of vaccine	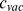	250,000^a^	27.64	Rp/dose
Costs of needle, syringe and swab	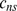	1,950^a^	0.22	Rp/patient
Cost for Physician	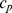	50,000^a^	5.53	Rp/Patient
Transportation cost of people received pre-exposure treatment*	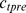	6,000^a^	0.66	Rp/visiting
Cost of water	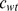	563^c^	0.06	Rp/per 30 liter/patient
Cost of soap	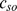	2,000^d^	0.22	Rp/patient
cost of antiseptic	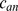	3,000^d^	0.33	Rp/patient
Proportion of human received immunoglobulin	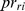	0.01^e^		
Price of Immunoglobulin	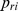	1,550,000^a^	171.37	Rp/dose
Number of doses of vaccine for post-exposure treatment	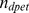	4^b^		doses/patient
Number of visits for receiving vaccination post-exposure treatment	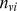	3^b^		visit
Transportation cost of people received vaccination post-exposure treatment**	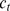	40,000^f^	4.42	Rp/visit
Daily wage	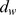	39,000^g^	4.31	Rp/day
Loss of working time for patient	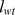	3^h^		day
Proportion of adult people received PET	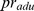	0.60^i^		

^a^ Public servants/veterinarians/internist involved in rabies control measures in the past;

^b^ WHO [6];

^c^ Market price of water in Kupang was approximately Rp 75,000 per 4,000 liter (Rp 18.75 per liter). We assumed that a patient will use the water about 2 litre per minute, so for 15 minutes wound cleaning (as recommended by WHO [6] and IHM [38]) the water needed was about 30 litre. Thus the price of water equal Rp 563 (Rp 18.75×30) per patient.

^d^ Assumption based on the market price in Flores in October 2011.

^e^ Bingham, [10];

^f^ Patients received immunoglobulin injection, and series of vaccine injections;

^g^ BPS [55];

^h^ Loss of working time for patient was set 3 days to visit the hospital 3 times to get PET;

^i^ WHO [58].

Transportation cost within the city since the people received pre-exposure treatment are public servants that working and living in the city.

Transportation cost from rural areas.

### Distribution of Costs

This study not only studied the total societal costs of rabies in Flores Island, it also evaluated the distribution of rabies control costs in terms of private and public costs [39]. Public costs are those that the Animal Health and Public Health departments incur, which are included in the local and/or national budgets. Private costs are those that dog owners and those exposed to the rabies virus incur.

The costs for dog owners include the loss of the value of dogs due to culling measures and income loss (opportunity costs) due to time lost while bringing dogs to be vaccinated and/or to catch their dogs. For exposed patients, costs include the opportunity costs for the patient and anyone accompanying the patient to get treatment and their transportation costs to a medical center for each treatment. Detailed components of public and private costs are shown in Table 5.

**Table 5:** The components of public and private costs of rabies control measures for different stakeholders.

	Stakeholders	Components
		1. Mass vaccination
		2. Culling of roaming dogs
	1. Agricultural Department	3. Dog-bite investigation
		4. Diagnostic testing of suspected rabid dogs
Public costs		5. Trace back investigation of human contacts
		6. Quarantine
	2. Public Health Department	1. Human rabies vaccines
		2. Immunoglobulin
		3. Syringe and needles
	3. Dog owners	1. The lost value of dogs due culling control measure
		2. Opportunity cost for the owner of vaccinated dogs
Private costs		3. Opportunity cost for the dog owners for their time investment to cull dogs
	4. Dog-bite patients	1. Opportunity cost for:
		• Patients
		• Caretakers
		2. Transportation of patients and caretakers

### Sensitivity Analysis

A sensitivity analysis was performed to identify those input parameters (Tables 2 and 4) that are highly influential to the costs of control measures. The sensitivity was based on a univariate analysis in which each parameter was increased and reduced by 10 percent of the default input values, as the others were held constant. The results of each change in parameter were compared with the results of the model outcome in the default situation to assess the impact of each parameter on the costs of rabies control measures.

## Results

### Total Costs of Control Measures

Total costs of rabies control measures during the study period (2000–2011) were estimated to be US$13.40 million, with an average of US$1.12 million (range: US$0.60–1.47 million) per year. The costs of control measures in dogs were about 28 percent higher than in humans. When ranked individually, regardless of control measures in dogs or humans, the costs of culling dogs were the highest, accounting for 39 percent of the total costs, followed by post-exposure treatment (35 percent), mass vaccination (24 percent), pre-exposure treatment (1.4 percent), and others (1.3 percent) (dog-bite investigation, diagnostic testing of suspected rabid dogs, trace-back investigation of human contact with rabid dogs, and quarantine of imported dogs) (Tables 6 and 7).

**Table 6:** Cost of Rabies control measures in dogs in Flores Island from 2000 to 2011.

Year	Costs of Rabies control measures in dogs (×1000 US$)						Total
	Massvaccination dogs	Cullingdogs	Biteinves-tigation	Diagnostictesting	Trace backinvestigation	Quarantine*	
2000	123.76	856.35	45.57	20.32	34.27	0.06	1,080.33
2001	125.23	797.18	22.28	9.94	16.80	0.06	971.49
2002	188.76	800.15	6.57	2.93	4.84	0.06	1,004.02
2003	293.22	136.51	0.73	0.33	0.29	0.06	431.13
2004	387.27	316.20	0.71	0.32	0.29	0.06	704.84
2005	395.76	465.28	0.61	0.27	0.15	0.06	862.14
2006	329.80	512.32	0.28	0.13	0.20	0.06	842.79
2007	186.61	715.57	0.24	0.11	0.20	0.06	902.78
2008	336.98	406.36	0.07	0.03	0.04	0.06	743.55
2009	363.34	172.09	0.16	0.07	0.07	0.06	535.80
2010	302.70	7.41	0.66	0.29	0.33	0.06	311.46
2011	186.93	3.36	0.92	0.41	0.62	0.06	192.30
Total	3,220.36	5,189.47	78.79	35.14	58.11	0.75	8,582.62

We assumed that the costs of quarantine were the same over time. This assumption based on the cost of quarantine control measure in 2011.

**Table 7:** Cost of Rabies control measures in humans.

Year	Costs of control measures in humans (×1000 US$)				Total	
	Pre-exposure treatment*	Post-exposure treatment				
		Wound cleaning	Rabies immunoglobulin	Humanrabies vaccine		
2000	15.32	1.50	3.63	370.89	391.34	
2001	15.32	0.32	0.77	78.35	94.75	
2002	15.32	0.62	1.50	153.50	170.94	
2003	15.32	0.59	1.44	147.11	164.46	
2004	15.32	0.75	1.82	185.90	203.79	
2005	15.32	1.89	4.58	467.49	489.28	
2006	15.32	1.37	3.71	379.21	399.61	
2007	15.32	2.01	5.18	529.21	551.72	
2008	15.32	2.12	5.17	527.63	550.24	
2009	15.32	2.31	5.57	569.16	592.37	
2010	15.32	3.01	6.42	655.90	680.65	
2011	15.32	2.19	4.96	506.25	528.72	
Total	183.85	18.68	44.75	4,570.60	4,817.89	

We assumed that the costs of pre-exposure treatment were the same over time.

This assumption is based on the costs of pre-exposure treatment control measure in 2011.

**Figure 1:**
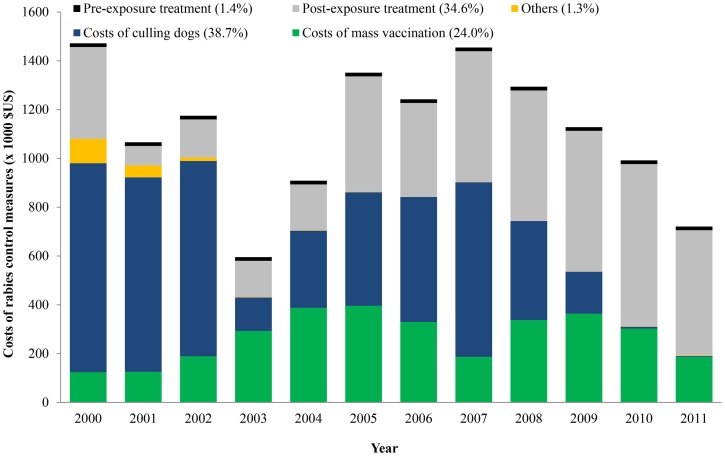
Distribution of costs by control measures and year in Flores Island.

The total costs of control measures fluctuated during 2000–2006, and tended to decrease in the last five years of the study period (Figure 1). The costs seemed to depend on the priority of rabies control measures applied. For example, in the first three years (2000–2002), the control program focused more on culling dogs, which is costly. Approximately 14 percent of the total dog population was culled at that time (Table 1). During 2008–2011, PET in humans dominated, at 41 percent to 71 percent of the total costs. In this context, the high proportion of PET costs in the total probably indicated not only a priority but also an increase in bite cases, and consequently, more PET.

### Costs of Control Measures in Dogs

Total costs of rabies control measures in dogs during the study period were estimated at US$8.58 million, with an average of US$0.72 million (range: US$0.19–1.08 million) per year (Table 6). Culling roaming dogs was the most costly measure, accounting for 60 percent of the annual costs of control measures among dogs, followed by mass vaccination of dogs (38 percent), bite investigation (1 percent), trace back investigation (0.7 percent), and diagnostic testing (0.4 percent). The quarantine of imported dogs accounted for almost nothing in total costs, a finding that could be underestimated because we assumed that the costs of quarantine remained the same throughout the study period.

The annual costs of mass vaccination of dogs were approximately US$268,360 (range: US$123,760–395,760), with a mean of US$2.49 per vaccinated dog. The price of vaccine contributed only 18 percent of the total vaccination costs of dogs. Other components were vaccinators, supervisors, meeting and training of temporary vaccinators, the information campaign, capital, and the opportunity costs of dog owners. In addition, the costs of mass vaccination of dogs increased from US$123,760 in 2000 to US$395,760 in 2005, and then fluctuated until 2011. This pattern indicates the government’s performance or commitment to control rabies through mass vaccination of dogs. Because of Indonesia’s autonomy system, the local governments of regencies provide budgets for vaccination control measures in dogs. Therefore, budget decisions regarding vaccination of dogs varied among Flores Island’s eight regencies, and the number of dogs vaccinated in each regency was not the same each year, depending upon budget allocations. Even when the central government (Agriculture Ministry of Indonesia) provides vaccines for dogs, regency budgets for training and hiring temporary vaccinators may determine the final vaccination coverage. This problem might contribute to the declining vaccination coverage in the last three years of the study period (2009–2011). Vaccination costs in 2011 were estimated to be two times lower than those in 2005 (Figure 1), as the vaccination coverage of registered dogs in 2011 (33 percent) was lower than in 2005 (69 percent) (Table 1).

Total costs of culling dogs were approximately US$5.2 million, with average annual costs about US$432,460 (range: US$3,360–856,350). The average costs per dog culled was estimated to be US$31.70. A large portion of these costs originated from the lost value of the dogs for the dog owners, which accounted for almost 100 percent of the total costs. Note that the annual costs of culling dogs were highest in the first year of the study period and then tended to fluctuate until reaching their lowest value in 2011 (Figure 1), which was about US$3,360.

The annual costs of diagnostic testing of suspected rabid dogs were calculated to be US$ 2,930 (range: US$30.00–20,320.00). The mean diagnostic costs per sample were estimated to be US$10.50. Interestingly, 53 percent of these costs were for shipping specimens to the rabies diagnostic laboratory and to correspondence of the diagnostic results. Specimens were sent to Maros, Sulawesi because there is no veterinary rabies diagnostic facility on Flores Island.

### Costs of Control Measures in Humans

The total costs of rabies control measures in humans were estimated to be US$4.82 million, with the largest portion being the PET costs. The pre-exposure treatment contributed only 3.8 percent of the total costs (Table 7). These costs were assumed to be constant every year since the number of people at high risk was stable over the years.

The annual costs of the PET were estimated to be US$386,170 (range: US$79,430–665,330), with most of the expenses related with the costs of a series of vaccine injections (99 percent).

The costs of PET for the first year of the study period were higher than for the next four years, because of a huge outbreak of rabies and a high number of people being bitten by suspected rabid dogs. The outbreak could be attributed to the higher number of roaming dogs. In 2001, the number of dogs decreased as a result of the culling control measure in 2000. The PET costs tended to increase, starting in 2001 (US$79,430) until 2010 (US$665,330) (Figure 1). The total costs of PET in 2010 were 8.4 times higher than those in 2001.

### Distribution of Costs

**Figure 2:**
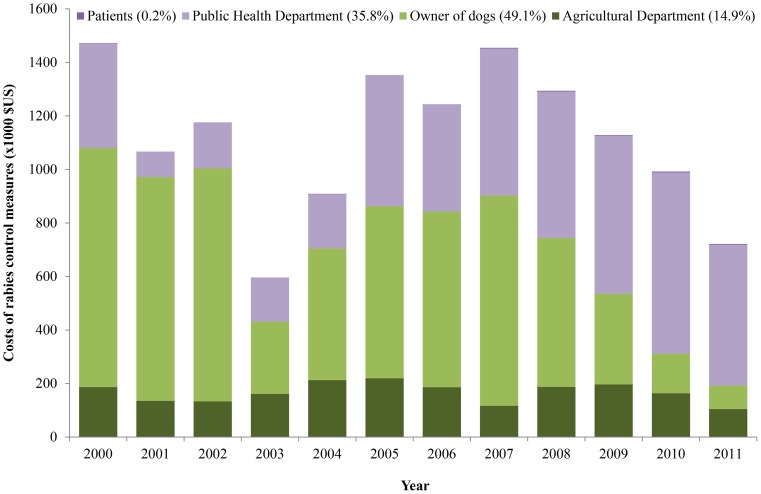
Distribution of Rabies control costs over different stakeholders and year in Flores Island.

Of the total costs of rabies control measures, public costs were higher (US$6.8 million) than private costs (US$6.6 million). The majority of public costs (71 percent) were incurred by the Public Health Department, which provided human vaccine and immunoglobulin for free to the local community. In addition, the annual proportion of public costs allocated by the government increased over time, with exception of 2000 (Figure 2). This increase reflects the fact that the number of people getting PET increased over the years. When the costs incurred by each stakeholder group during the study period were ranked, the total costs for dog owners was the highest portion, or about 49 percent of the total societal costs, followed by costs incurred by the Public Health Department (36 percent), the Agricultural Department (15 percent), and patients (0.2 percent) (Figure 2).

### Sensitivity Analysis

The total costs of rabies control measures were most sensitive to the dog value. An increase or decrease of the dog value by 10 percent resulted in a 4 percent change in total costs. Other input parameters that influenced the total costs in our analysis were the price of human rabies vaccine and the number of vaccine doses in humans; a 10 percent increase or decrease in these parameters, resulted in both cases in a 2 percent change in total costs. Other inputs contributed to changes in the default total costs of less than 2 percent.

## Discussion

A deterministic economic model was developed to evaluate the costs of rabies control on Flores Island during 2000–2011. With this model, we calculated the total costs of rabies control measures as they were carried out on Flores Island, by integrating available epidemiological and economic data, scientific literature, and information from experts in rabies control measures. The results are an estimation because some inputs (price of vaccine, immunoglobulin) were uncertain in the analysis. The described analysis is an ex-post analysis. However, the developed calculation model is set up in such a way that it can be used to predict the costs of future rabies control programs (ex-ante analysis), not only for Flores Island but also for other regions or countries.

Some limitations of this study may have led to over- or under-estimation of the total costs of control measures. For example, the costs of control measures in humans might have been overestimated because we assumed all people were injected with four doses, despite the fact that the dog-bite patients might receive fewer than four doses in reality. Moreover, the epidemiological surveillance and research costs were not considered in the analysis because of a lack of data. Also, the costs for diseased livestock and human patient cases were not included, which may have led to an under-estimation of the costs of rabies. In none of the regencies in Flores cases of rabies in livestock have been reported, although the Husbandry Department of East Nusa Tenggara province provides the livestock owners a format to report any rabies cases in the livestock. So this omission is, most probably not related with a large under-estimation of the costs of rabies. However, no data were available on the number of patients that were hospitalized due to rabies. Unfortunately, therefore, we were not able to make an estimation of the costs for human patients. Despite these limitations, the estimate made in this study illustrates the economic burden of rabies control measures for all stakeholders on Flores Island, Indonesia as realistic as possible. Our results show that the costs of culling roaming dogs were the highest portion (39 percent) of the total costs, with average costs per dog culled at US$31.70. This finding contrasts with other studies that found the highest portion of costs were for PET [40], [41], [42], [43]. Knobel et al. [42] studied the economic burden of rabies at the regional level in Asia and Africa and found that the highest portion (83 percent) of the total control budget was allocated to PET. The World Health Organization, as cited by Voelker [43], estimated the costs of rabies in Asia to be about US$560 million every year, with the largest portion spent on PET. The proportion of costs of culling roaming dogs in Asia and Africa was lower than in our findings, with the average cost per dog culled at US$5 [42]. The difference is due to the value of dogs, which their analysis ignored. In our analysis, the largest part of the costs of culling dogs was the value of the dogs. Ignoring the value of dogs would significantly reduce the contribution of the costs of culling dogs to only 1.6 percent of the total estimated costs.

The second largest costs for rabies control measures were those of PET, an average of US$178 per patient. The expensive human rabies vaccine and/or immunoglobulin [24] and the high number of the dog-bite patients receiving PET [44] contributed to the high PET costs in this study. Our findings were a little bit higher than those in Thailand [25], but lower than those in the United States [17]. In Thailand, the costs of PET were estimated to be US$135–154 per patient [25], while the costs in the United States were estimated to be US$ 1,707 per patient [17]. This disparity is caused by differences in prices of human vaccine, immunoglobulin, transportation costs, labor costs, scheduled vaccine, and the type of vaccine used. For example, in the United States, human diploid cell vaccine was used with a cost range of US$80–483 per dose [45], while purified chick embryo rabies vaccine was used in Thailand, with a cost range of US$13–14 per dose [25].

This study also found that the annual costs of PET increased in the last seven years of the study period, which reflects the increased number of dog-bite patients who received PET (Figure 2) as the vaccine became more widely available. PET for humans is an effective but costly way to prevent clinical problems with rabies but does not provide a permanent solution to rabies in the future. The costs of PET (US$178 per patient) equals approximately 41 times the daily wage of people in Flores Island. This finding is higher than in Asia (US$49.41 equivalent to 14 times daily wage) and Africa (US$39.5721 equivalent to 21 times daily wage) [18].

The current control measures in the dog populations were not successful in reducing the number of human bite cases by suspected rabid dogs and rabies as such is still endemic in Flores Island. Some explanations that may contribute to this situation; (1) there was no island-wide dog vaccination campaign as, for instance, carried out on Bali Island [14] due to lack of resources; (2) the locally produced killed rabies vaccine has a relatively low duration of immunity and booster vaccination is recommended at three months, but rarely implemented [46]; (3) in addition, the actual number of dogs in Flores Island is unknown. The number of dogs in this study is based on the administration record of Animal Husbandry Department East Nusa Tenggara Province. These registered data were submitted annually by eight Regency Husbandry Department in Flores Island. These data underestimate the actual number of dogs present since the data are based on the recording during the vaccination campaign. In case the dog owners and their dogs were not at home at the moment of the vaccination campaign, the dogs were not registered (Dr. Siko, Personal communication). Therefore, the vaccination coverage level of >70% during the year 2004–2006 as indicated by Table 1 was overestimated.

Furthermore, of the total dogs registered, the percentage of vaccinated dogs was less than 100%. There are two possible reasons that could explain this situation as described in detail by [14], [47], [48], [49], [50]. The first reason is related to the young age of the dogs at the time of the vaccination campaign. Generally it is recommended by vaccine manufacturers not to vaccinate dogs which are younger than 3 months of age. The proportion of this cohort of young dogs could reach up to 39% of the total population dogs [50]. The second reason is related to the inaccessibility of free roaming dogs as in the case of Bali Island, Indonesia [14], which might be due to a lack of willingness by the dog owners to participate in the vaccination program.

The culling program of dogs in infected areas failed to prevent the virus spreading throughout the island since not all local people were willing to participate in culling dogs. Only a few local people (approximately 5–10 people in each village) joined as volunteers in the culling of dog procedure.

The annual total costs of control measures in humans increased over the years, a finding that contrasts with other studies in different countries. In many countries, rabies control measures in dogs have substantially reduced the costs of PET in humans because fewer people seek PET [13], [51]. Glosser et al. (1970) reported that an increased number of vaccinated dogs, combined with culling stray dogs decreased the number of people bitten by a suspected rabid dog, resulting in reduced numbers of people getting vaccine or immunoglobulin anti-rabies by 91 percent (from 1,116 in 1966 to 170 in 1968). In addition, Cleaveland et at. [13] studied a rabies vaccination campaign of dogs in rural Africa and found that vaccination coverage of 60–70 percent of the dog population significantly reduced the PET in humans. This would imply that rabies control in the dog population significantly contributes to a reduction of the economic burden caused by expensive PET.

This is the first study to consider the value of culled dogs in rabies control. This factor was included because dogs have an economic value and are culturally very important for the local society [9], [23], [30]. Dog meat is a popular menu item in certain traditional events. Besides being a source of protein, dogs also guard property and chase away wild animals (wild pigs, mice, and monkeys) that destroy farmers’ crops. As a consequence good guard dogs are highly priced at the traditional markets [23].

Therefore, culling as a control measure might be less acceptable for a local community because of ethical, social, and economic reasons. In this context, the World Organization for Animal Health [52] does not recommend culling dogs as priority in control and eradication of rabies. There is no evidence that culling dogs alone significantly contributes to a reduction of the spread of rabies [6]. Therefore, the local government of Flores Island reduced the culling of dogs over the years, which lowered the annual costs of that control measure.

Our results demonstrate that the rabies control measures were costly to society. Optimization of the current control measures could reduce the economic burden of rabies in the future. An economic study that weighed the trade-off between controlling rabies in dogs and PET in humans is needed to determine if more control among dogs would be cost beneficial. This study’s results could provide baseline data for additional effectiveness studies.

### Conclusion/Significance

This study shows a generic and transparent way to calculate the societal costs of rabies in a certain region. Rabies has a large economic impact on government and the dog owners of Flores Island. Control of rabies by culling dogs is relatively costly for the dog owners in comparison with other control measures. Providing PET is an effective way to prevent rabies casualties in humans, but is costly for the government, without providing a permanent solution for rabies control in the future. The developed model can be used for future economic ex-ante and ex-post analyses on rabies control.
